# Insulin-like growth factor-2 regulates basal retinal insulin receptor activity

**DOI:** 10.1016/j.jbc.2021.100712

**Published:** 2021-04-26

**Authors:** Sergey N. Zolov, Hisanori Imai, Mandy K. Losiewicz, Ravi S.J. Singh, Patrice E. Fort, Thomas W. Gardner

**Affiliations:** 1 Department of Ophthalmology & Visual Sciences, University of Michigan Medical School, Ann Arbor, Michigan, USA; 2 Department of Internal Medicine, University of Michigan Medical School, Ann Arbor, Michigan, USA; 3 The Division of Pulmonary & Critical Care Medicine, University of Michigan Medical School, Ann Arbor, Michigan, USA; 4 Department of Ophthalmology, Kobe University Medical School, Kobe, Japan; 5 Retina Associates, Kansas City, Missouri, USA; 6 Department of Molecular & Integrative Physiology, University of Michigan Medical School, Ann Arbor, Michigan, USA

**Keywords:** insulin-like growth factor-2, insulin receptor, insulin, retina, diabetes, HRP, horseradish peroxidase, IGF-1, Insulin-like growth factor-1, IGF-2, Insulin-like growth factor-2, IGF1R, Insulin-like growth factor-1 receptor, IGFBP, Insulin-like growth factor binding protein, IR, Insulin receptor, PY, phosphorylation

## Abstract

The retinal insulin receptor (IR) exhibits basal kinase activity equivalent to that of the liver of fed animals, but unlike the liver, does not fluctuate with feeding and fasting; it also declines rapidly after the onset of insulin-deficient diabetes. The ligand(s) that determine basal IR activity in the retina has not been identified. Using a highly sensitive insulin assay, we found that retinal insulin concentrations remain constant in fed *versus* fasted rats and in diabetic *versus* control rats; vitreous fluid insulin levels were undetectable. Neutralizing antibodies against insulin-like growth factor 2 (IGF-2), but not insulin-like growth factor 1 (IGF-1) or insulin, decreased IR kinase activity in normal rat retinas, and depletion of IGF-2 from serum specifically reduced IR phosphorylation in retinal cells. Immunoprecipitation studies demonstrated that IGF-2 induced greater phosphorylation of the retinal IR than the IGF-1 receptor. Retinal IGF-2 mRNA content was 10-fold higher in adults than pups and orders of magnitude higher than in liver. Diabetes reduced retinal IGF-2, but not IGF-1 or IR, mRNA levels, and reduced IGF-2 and IGF-1 content in vitreous fluid. Finally, intravitreal administration of IGF-2 (mature and pro-forms) increased retinal IR and Akt kinase activity in diabetic rats. Collectively, these data reveal that IGF-2 is the primary ligand that defines basal retinal IR activity and suggest that reduced ocular IGF-2 may contribute to reduced IR activity in response to diabetes. These findings may have importance for understanding the regulation of metabolic and prosurvival signaling in the retina.

Peptide growth factors are essential for the proliferation and differentiation of neuroprogenitor cells and for survival of fully differentiated retinal neurons, particularly under stress conditions (1). The evolutionarily conserved roles of insulin-like growth factors (IGFs) and insulin predate the development of the pancreas (2), and these ligands have prominent effects on retina and brain development and function. For example, deletion of the insulin receptor (IR) causes morphologic defects in the eye and brain of zebrafish (3) and *Drosophila* (4), and deletion of the mouse insulin responsive substrate gene, *Irs-2*, impairs retinal ganglion cell and photoreceptor maturation (5). The rat retina exhibits a high level of basal IR kinase activity equivalent to that in the liver of fed animals but does not fluctuate with feeding or fasting (6). We also showed that periocular insulin administration activates both the retinal IR and the IGF-1 receptor (IGF1R) (6). These findings strongly support an important role of the insulin/IGF ligand/receptor pathways in the developing and adult nervous system.

Insulin-deficient diabetes causes accelerated apoptosis of inner retinal neurons (7) in parallel with reduced basal activity of the IR → PI3-kinase → Akt → p70^S6 kinase^ pathway, independent of IGF1R kinase activity (8, 9, 10, 11). Insulin and IGF-1 are neurotrophic for retinal neurons *via* activation of the Akt pathway, a pathway specifically impaired by diabetes in the retina and metabolic stress conditions in cultured neurons (12, 13, 14). Systemic, intravitreal, and subconjunctival administration of insulin restores prosurvival IR and Akt kinase activities and reduces retinal cell death associated with diabetes (9, 11). However, it is unknown how the *basal* IR activity in normal adult animals is determined and how diabetes impacts this regulation. We first asked if the basal set point of retinal IR activity might be regulated by plasma insulin binding to retinal receptors. However, feeding and fasting does not change retinal IR activity as it does in the liver (6). Thus, we reasoned it was possible that the stable high basal retinal IR activity involves locally derived agonist ligand(s).

*Igf-1* and *Igf-2* are expressed primarily in the liver and brain during development. Adult rats continue to express *Igf-1* and *Igf-2* in the brain with persistent transcriptional activity, whereas liver production of IGF-2 is nil (reviewed in (15)). Lofqvist *et al.* (16) showed that the IGF/insulin family of ligands and receptors is abundantly expressed in the mouse retina. Igf2 mRNA is 100- to 1000-fold more abundant than Igf1 or insulin (Ins) mRNA, and Igf1 receptor (Igf1R) is more abundant than the IR. Supporting an important role of IGF-2 in fully differentiated neuronal cells, *Igf2*-null mice have increased susceptibility to sciatic nerve transection (17). Of note, the growth-promoting effects of IGF-2 during development are mediated largely *via* the IR (18), and the retinal IR is highly sensitive to IGF-2 (reviewed in (19)).

Taken together, these data led us to hypothesize that IGF-1 and/or IGF-2 may be important endogenous ligands of the retinal IR and play a critical prosurvival role in retinal neurons, a function disrupted by diabetes. Initial studies revealed persistent Igf1, Igf2, Ins, and IR transcript levels in the retina and liver during postnatal development and experimental diabetes. After assessing the basal retinal IR kinase activity, we demonstrated that intravitreal administration of a neutralizing IGF-2 antibody specifically reduces retinal IR kinase activity in normal rats. Moreover, we observed that diabetes reduces both retinal Igf2 mRNA content and IGF-2 protein content in the vitreous. Finally, we showed that intravitreal administration of rhIGF-2 restores retinal prosurvival signaling cascades in diabetic rats. Collectively, these findings provide novel insights into the regulation of prosurvival signaling pathways in the retina and how they are compromised by diabetes.

## Results

### Retinal IGFs and insulin receptor expression during ontogeny and diabetes

**Figure 1:**
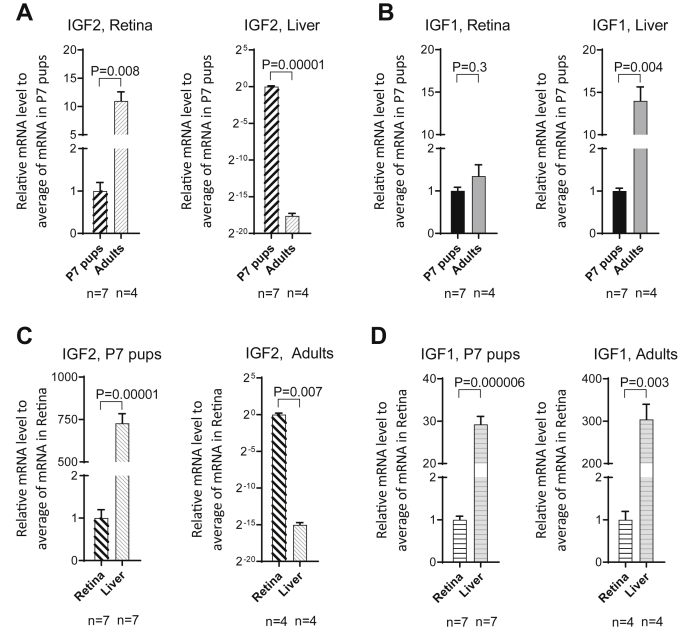
***IGF-1* and *IGF-2* transcript levels in retina and liver vary during ontogeny.***A*, *IGF-1* and *IGF-2* mRNA contents of retina and liver were compared by quantitative PCR in P7 pups and healthy adult male rats. Data analyzed by *t* tests. *B*, retina and liver *IGF-1* expression were compared during postnatal development. *C*, *IGF-2* expression in P7 liver in comparison to adult retina. *D*, comparison of *IGF-1* content in retina and liver at P7 and adult rats. IGF-1, insulin-like growth factor-1; IGF-2, insulin-like growth factor-2.

Lofqvist *et al.* (16) reported the high relative expression of retinal *Igf*-1/2 mRNA’s compared with insulin mRNA in normal mouse retina, but little is known about their expression and potential role in the adult mammalian retina. Previous studies have shown that IGF-1 and IGF-2 are important for cell growth (20), so transcript levels were compared by qPCR in P7 pups and adult rats. Figure 1*A* shows that *Igf2* mRNA content is 10-fold higher in adult retina than in postnatal retina; moreover, adult liver *Igf2* expression is significantly less than in pups. These data are consistent with reports of high *Igf2* content in adult brain compared with fetal brain (21, 22). By contrast, liver *Igf1* mRNA was higher in adult than P7 pups, and retinal *Igf1* content was equivalent in postnatal and adult eyes (Fig. 1*B*). Tissue-specific comparisons (Fig. 1, *C* and *D*) further reveal variations in *Igf2* expression during ontogeny. There is 750 times more *Igf2* mRNA in P7 liver *versus* retina, but adult retina *Igf2* is substantially higher than in liver. By contrast, *Igf1* content in P7 liver is 30-fold higher than in retina, and in adult rats, this difference further increases by a factor of 10 times. These results reveal that the adult retina expresses abundant IGF-1 and IGF-2, which likely function in an autocrine or paracrine manner (15).

**Figure 2:**
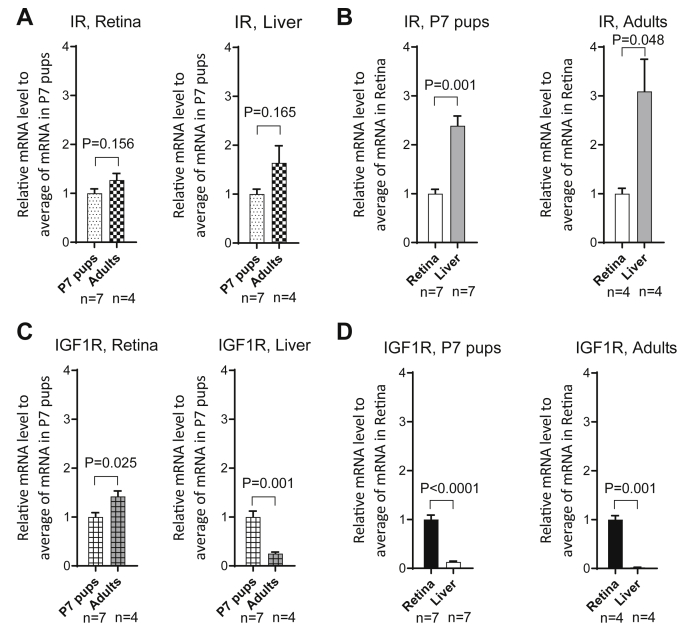
**Insulin receptor and IGF-1 receptor transcript levels during postnatal development.***A*, retinal and liver *INSR* mRNA levels were compared by quantitative PCR in P7 pups and adult rats. Data analyzed by *t* tests. *B*, *INSR* mRNA content in liver and retina in P7 pups and adult rats. *C*, retinal *IGF1R* mRNA content in the liver and retina of P7 pups and adult rats. *D*, *IGF1R* mRNA content in retina and liver in P7 pups and adult rats. IGF-1, insulin-like growth factor-1; IGF1R, insulin-like growth factor-1 receptor.

Analysis of IR expression patterns (Fig. 2*A*) reveals that (1) retinal and liver IR mRNA contents are similar between P7 and adult animals and (2) IR mRNA content is 2- to 3-fold higher in liver than retina at both P7 and adult stages (Fig. 2*B*). Conversely, while there is a modest increase in Igf1R mRNA in adult compared with P7 retina, there is a 75% reduction in adult liver, leading to retinal expression being orders of magnitudes higher than liver in adulthood (Fig. 2, *C* and *D*). Collectively, these data demonstrate a high level of retinal IR and IGF1R expression in adult retina.

**Figure 3:**
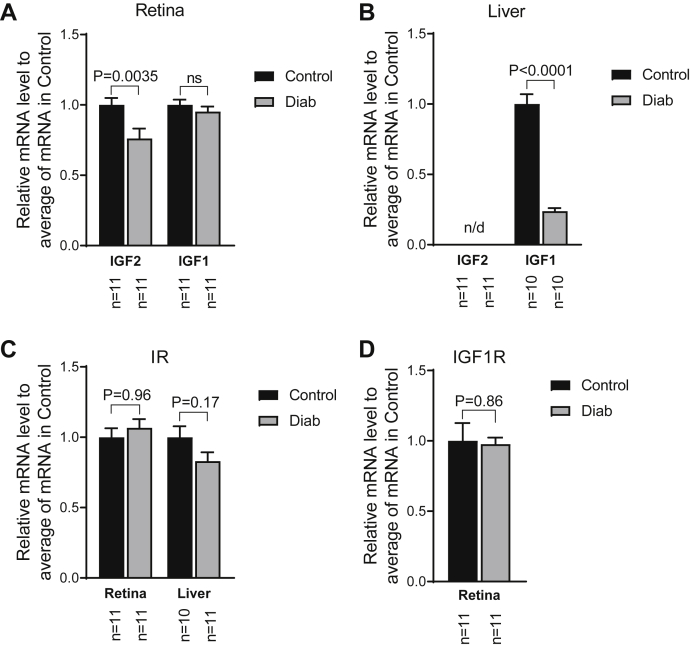
**Diabetes reduces *IGF-2* mRNA in the retina and *IGF-1* mRNA in the liver.***A*, effects of 4 weeks of diabetes on retinal *IGF-2* and *IGF-1* mRNA content as measured by quantitative PCR. Data analyzed by *t* tests. *B*, effects of diabetes on *IGF-2* and *IGF-1* expression in liver. *C*, effects of diabetes on *INSR* expression in retina and liver. *D*, effects of diabetes on *IGF1R* content in retina. IGF-2, insulin-like growth factor-2; IGF1R, insulin-like growth factor-1 receptor.

To assess the mechanisms of reduced retinal IR kinase activity in diabetes, qPCR was performed for *Insr* and *Igf*1R and the associated ligands in control and age-matched rats after 4 weeks of diabetes (Fig. 3, *A*–*D*). *Igf-2* but not *Igf-1* mRNA content decreased in diabetic rat retinas; *Igf-1* mRNA decreased in the diabetic rat liver, but *Igf2* was not detected in this tissue. *Insr* and *Igf1r* mRNA contents were not changed by diabetes in either tissue. Body weights and blood glucose values are shown in Fig. S1.

### Analysis of retinal IR ligands

We previously showed that insulin, IGF-1, and IGF-2 can trigger IR autophosphorylation in *ex vivo* retinas (6), but there remained questions relative to the endogenous ligand(s) of the retinal IR *in vivo*. In the current study, we used a series of complementary approaches to examine the role of pancreas-derived insulin in the regulation of retinal IR activity.

**Figure 4:**
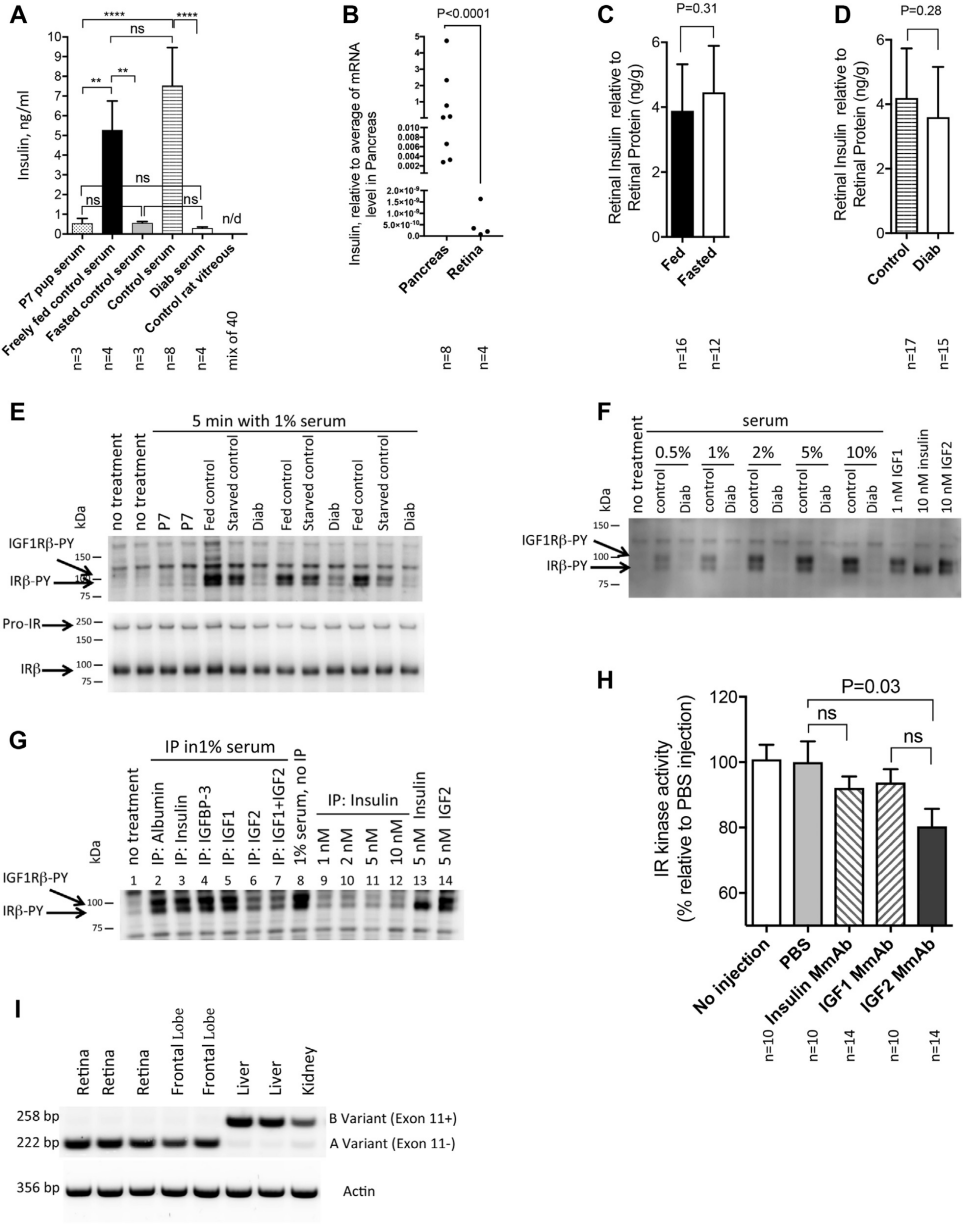
**IGF-2 is an endogenous ligand for the retinal insulin receptor.***A*, serum insulin concentrations vary with feeding and fasting and with diabetes, but insulin is not detectable in vitreous gel. Insulin concentrations were determined by radioimmunoassay (RIA) in the serum of control P7 pups and control adult rats under fed and fasted conditions and in diabetes conditions (4 weeks) and in their age matched control. Control rat vitreous insulin concentrations were also determined by RIA. Data analyzed by one-way ANOVA (F (_5,17_) = 25.75, *p* < 0.0001) followed by *post hoc* Tukey's multiple comparisons test, with a single pooled variance (∗∗*p* = 0.005, ∗∗∗∗*p* < 0.0001), n/d, not detected. *B*, pancreas and retinal INS mRNA content in normal rats. Insulin mRNA levels in the pancreases, retinas of control rats under fed conditions were assessed by qPCR. Error bars, SD. Data analyzed by *t* tests. *C* and *D*, retinal insulin concentrations are unchanged by feeding or fasting or by insulin-deficient diabetes. Retinas were removed, frozen, and insulin content relative to total protein was quantified in fasted and fed adult control rats (*C*) and in control and 6 weeks duration diabetic (*D*) Sprague-Dawley rats by RIA. Error bars, SD. Data analyzed by *t* tests. *E*, serum from fed animals stimulates IR-ß autophosphorylation. Serum (1%) from fed P7 rats, fed or fasted adult control rats, and diabetic rats was used to treat R28 cells, and phosphorylation of IGF1Rß and IR-ß was assessed by immunoblotting. Samples were normalized to equal protein content and total IR-ß was used to confirm equivalent loading. *F*, dose-dependent effects of control and diabetic rat serum on IGF1Rß and IR-ß autophosphorylation in R28 cells using 0.5% to 10% serum for 5 min. Human insulin, rhIGF-1, and rhIGF-2 were used as positive controls. Proteins were analyzed by SDS-PAGE and PY blotting, normalized to equal protein content. *G*, depletion of IGF-2 from control rat serum specifically reduced the ability to phosphorylate IR and IGF1R in R28 cells. Rat serum was treated with antibodies to albumin, IGFBP3, insulin, IGF-1 and IGF-2 (samples # 2–7). Sample # 8 is control R28 cells treated with 1% rat serum. Samples # 9 to 12: control Insulin immunoprecipitation of 1, 2, 5, and 10 nM diluted the cell culture medium. Samples 13 is cell culture medium with 5 nM insulin and sample 14 is 5 nM IGF-2 without immunodepletion. Samples were normalized to equal protein content. *H*, IGF-2 immunodepletion reduces retinal IR kinase activity. Normal adult rats were injected intravitreally with PBS or antibody to insulin, IGF-1 or IGF-2; retinas were harvested after 60 min and assessed for IR kinase activity as described (11). Data analyzed by a one-way ANOVA (F (_4,53_) = 3.094, *p* = 0.0231) followed by *post hoc* Tukey's multiple comparisons tests. Error bars, SEM. Results representative of five independent experiments. *I*, normal retina expresses only the exon 11-splice form. Insulin receptor splice variants were compared by qPCR from normal adult rat retina, brain, and liver. Results were in duplicates. IGF-2, insulin-like growth factor-2; IGF1R, insulin-like growth factor-1 receptor; IR, insulin receptor; PY, phosphorylation.

First, serum insulin concentrations were determined in P7 pups and in control and diabetic adult rats. Figure 4*A* shows the expected 10-fold greater serum insulin concentration in fed control *versus* paired animals after an overnight fast. Likewise, 4 weeks of insulin-deficient diabetes resulted in nearly complete loss of insulin, near the limits of assay sensitivity (0.2 ng/ml). No insulin could be detected in vitreous fluid of healthy rats.

A second approach compared pancreatic and retinal insulin mRNA content (Fig. 4*B*), and as expected, pancreas insulin mRNA content was six orders of magnitude greater than in retina. The wide variation in insulin mRNA content is likely because of the heterogeneous distribution of islets in the rat pancreas (23). We cannot exclude the possibility that the low level of retinal insulin gene expression contributes sufficient peptide ligand to activate the retinal IR. We further determined that retinal insulin protein content did not vary with overnight fasting or diabetes (Fig. 4, *C* and *D*). Our values of approximately 4 ng insulin/g retinal tissue are equivalent to those reported by LeRoith *et al.* (24) for brain and suggest a consistent level in the CNS. Together, these results distinguish the regulation of stable basal retinal IR activity from the fluctuating IR activity in peripheral tissues and suggest the possibility that additional ligands may regulate retinal IR activity.

The preceding studies led us to employ a third approach using a cell culture system to examine the effects of physiologic fasting/feeding and streptozotocin-induced insulin-deficiency on IR phosphorylation (PY)in R28 retinal neurons, an established model for signaling studies (14, 25). We compared the effects of serum from fed, fasted, and diabetic rats on IR and IGF1R autophosphorylation and used immunodepletion techniques to assess the contribution of specific components of serum on receptor PY. Compared with untreated cells, 1% serum from fed rats induced prominent IR and IGF1Rß tyrosine PY that was approximately 50% greater than that from serum of rats fasted overnight (Fig. 4*E*) and increased in a concentration-dependent manner from 0.5% to 10% (Fig. 4*F*). By contrast, cells treated with serum from insulin-deficient diabetic rats did not show any increase over control cells, indicating the diabetic serum lacked an important soluble factor.

To further determine the ligand(s) responsible for the effect of the serum, immunodepletion of albumin, insulin, insulin-like growth factor binding protein-3 (IGFBP3), IGF-1, and IGF-2 was performed from control serum (Fig. 4*G*, samples # 2–7). Anti-insulin antibodies in 1% serum (sample # 3) in an amount sufficient to immunodeplete 10 nM insulin did not decrease IR autophosphorylation, thus pointing to another ligand. Depletion of IGF-2, however, reduced IR and IGF1R-PY by approximately 50%, but dual depletion of IGF-1 and IGF-2 had no additive effect. Depletion of albumin, IGFBP3, and IGF-1 did not diminish IR or IGF1R-PY specific immunoreactivity. The insulin, IGF-1, and IGF-2 antibodies were specific for each ligand. Anti-insulin antibodies blocked the IR PY when insulin up to 10 nM was added directly to the medium (samples 9–12 in Fig. 4*G*). The last two lanes of Figure 4*G* show that equimolar IGF-2, and insulin have equivalent effects on IR-PY content. These data demonstrate the specific effect of IGF-2 on retinal IR PY. Stimulation of R28 retinal neuronal cells showed a concentration-dependent increase of IR and IGF1R PY in response to serum from control but not diabetic animals (Fig. 4*F*). Taken together, these studies further support the role of IGF-2 as a ligand of retinal IR and IGF1R.

The fourth experiment employed intravitreal injections of multiple neutralizing antibodies against insulin, IGF-1, and IGF-2 to determine the endogenous retinal IR ligand(s). Figure 4*H* shows that compared with uninjected eyes and eyes that received intravitreal PBS, insulin and IGF-1 monoclonal antibodies did not significantly reduce IR kinase activity, whereas the IGF-2 antibody significantly reduced IR kinase activity by 20%. Results are representative of five independent experiments. These acute experiments avoid potential compensation that might occur from genetic deletion of retinal IGF-2. These data indicate that IGF-2 is the predominant endogenous ligand contributing to the basal activity of the retinal IR.

Fifth, the affinity of the IR for insulin and IGFs depends on the mRNA splice variants expressed (26). Therefore, IR splice variants were compared by PCR from normal adult rat retina, brain, and liver. Figure 4*I* shows the liver and kidney express only the B (exon 11+) splice variant, whereas retina and brain frontal lobe express only the A (exon 11-) form, which is more sensitive to IGF-2 (6). These findings, combined with the 100- to 1000-fold higher level *IGF-2* mRNA expression compared to insulin or *IGF-1* (16), suggest autocrine/paracrine activation of the retinal IR by IGF-2, consistent with the high basal activity independent of feeding and fasting.

### Patterns of retinal insulin receptor phosphorylation induced by IGF-2

**Figure 5:**
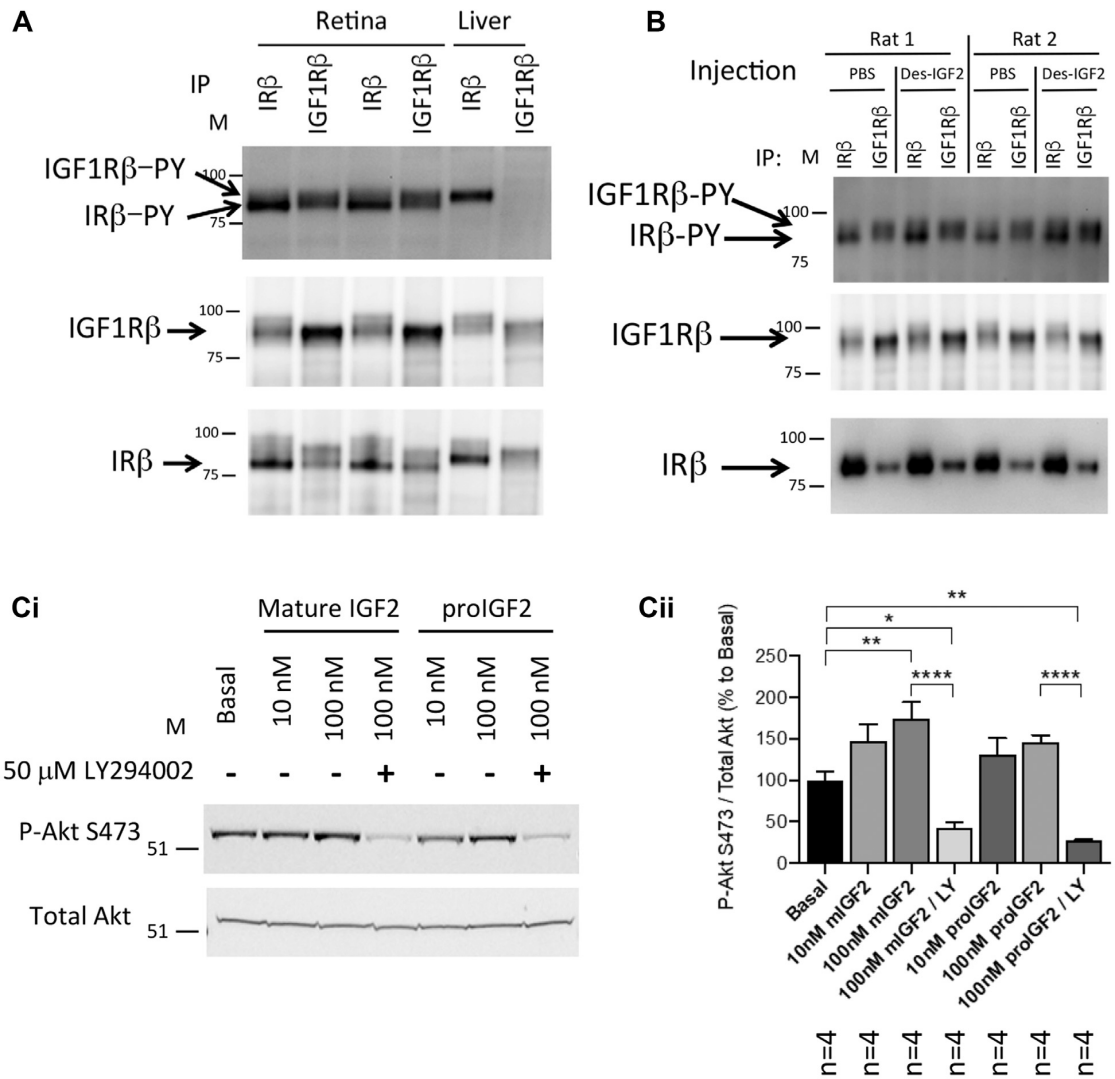
**IGF-2 increases *ex vivo* retinal insulin receptor autophosphorylation and Akt phosphorylation.***A*, unstimulated normal rat retina and liver lysates from normal fed male Sprague-Dawley rats were immunoprecipitated with IR-ß and IGF1R antibodies and assessed for phosphotyrosine (*top panel*). Membranes were stripped and re-probed for IGF1Rß (*middle*) and IR-ß content (*bottom*). *B*, normal Sprague-Dawley rats received a single intravitreal injection with 5 μl of PBS or 1 μM rhDes[1–6]-IGF-2 and retinal lysates were assessed for IGF1R-PY and IRß-PY. Membranes were stripped and re-probed for IGF1Rß (*middle*) and IR-ß content (*bottom*). *C*, IGF-2 and pro-IGF-2 activate retinal Akt in a PI3-kinase dependent manner. *Ex vivo* rat retinas from normal Sprague-Dawley rats were pretreated with or without 50 μM LY294002 for 15 min then treated with mature rhIGF-2 or rhpro-IGF-2 treatment for an additional 5 min (*Ci* – a representative Western blot of total and phospho-Akt). Lysates were assessed for Akt Ser473 phosphorylation was normalized to total Akt content. Data (*Cii*) analyzed by one-way ANOVA (F (_6,21_) = 15.00, *p* < 0.0001) followed by *post hoc* Tukey's multiple comparisons test, with a single pooled variance (∗*p* < 0.05, ∗∗*p* < 0.01, ∗∗∗∗*p* < 0.0001). IGF-1, insulin-like growth factor-1; IGF-2, insulin-like growth factor-2; PY, phosphorylation.

We showed previously that insulin and IGF-2 activate the IR in normal *ex vivo* rat retinas (6). Here, we compared basal *in vivo* IR and IGF1R–specific autophosphorylation by PY blotting after immunoprecipitation (Fig. 5*A*). As we and others (9, 27, 28) have reported, both receptors form hetero-oligomers which leads to their co-immunoprecipitation. They can be individually assessed because the IR-ß subunit electrophoretically migrates slightly faster than IGF1Rß. As reported previously (6), the IR-ß from liver (11+ exon) migrates more slowly than that from retina (11− exon), and the adult rat liver expresses essentially no IGF1Rß protein. In addition, we injected Des[1–6]-IGF-2 lacking the IGF binding protein domain (29) intravitreally in normal rats and assessed receptor autophosphorylation. The pattern shown in Figure 5*B* demonstrates a 2-fold greater increase in retinal IR-ß autophosphorylation than IGF1Rß autophosphorylation in response to IGF-2 *versus* PBS. Thus, the IR is a target for IGF-2 in the retina.

IGF-2 activates IRs in adults (30); thus, we examined the effects of recombinant human mature and pro-IGF-2 in *ex vivo* retinas. Both IGF-2 forms caused concentration-dependent increases in Akt Ser473 phosphorylation, and the effects were blocked by pretreatment with LY294002 (Fig. 5*Ci* and *ii*). These data further confirm the retina IR responds to IGF-2 stimulation *via* a PI3-kinase dependent pathway.

### Retinal and vitreous IR ligand expression during diabetes

**Figure 6:**
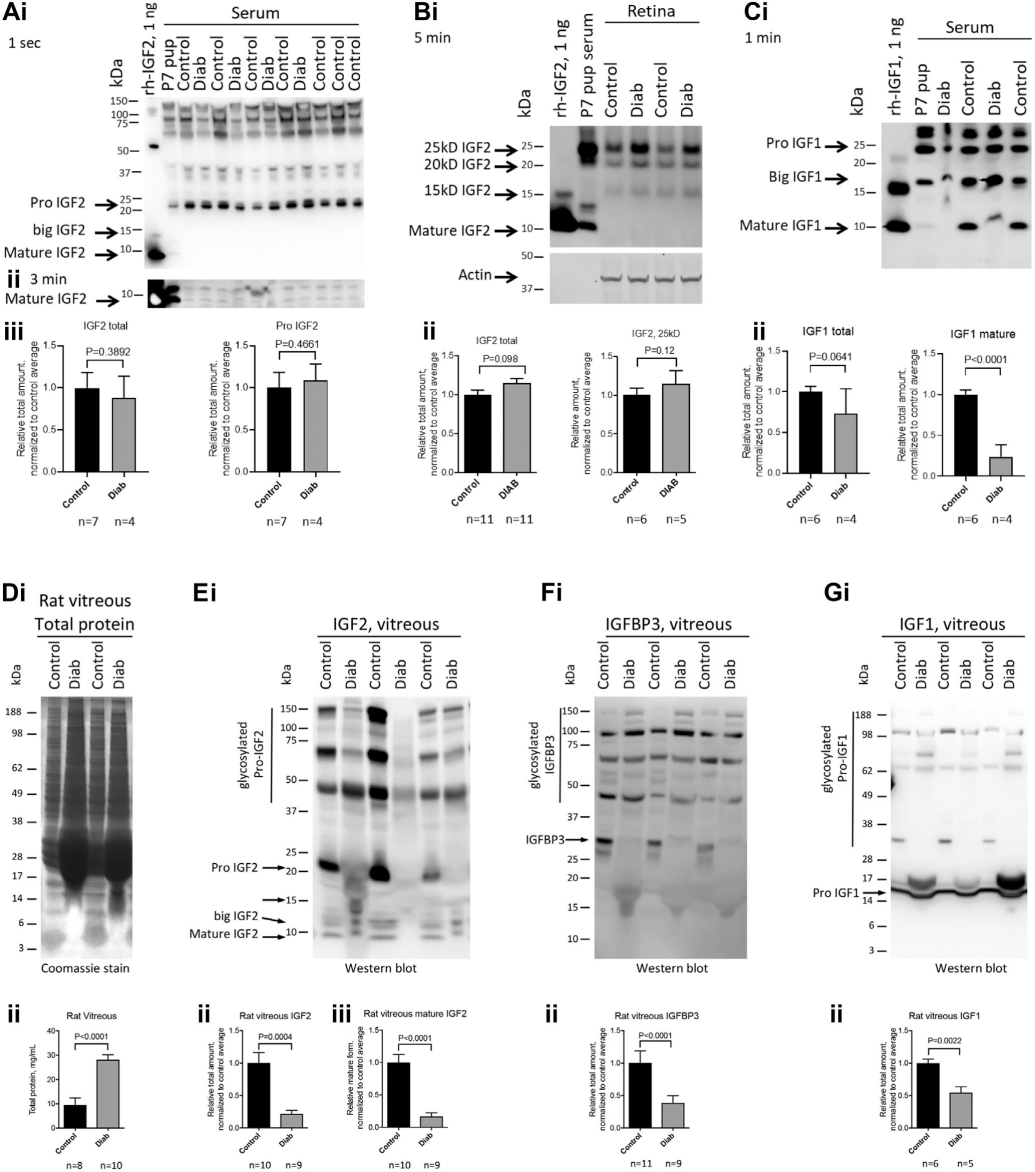
**Retinal and vitreous insulin receptor ligand expression during diabetes.***A*, serum IGF-2 content is reduced in 4-week diabetic rats as assessed by immunoblotting under nonreducing conditions as described (27); representative immunoblots exposed for 1 s (*Ai*), 3 min (*Aii*), and quantification of 1 s exposed immunoblot (*Aiii*). *B*, retinal IGF-2 content in control and diabetic animals relative to 1 ng rh-IGF-2 and P7 pup serum (*Ai*) and quantification (*Aii*). *C*, relative serum IGF-1 content in control and diabetic rat serum as determined by immunoblotting and quantification (*Aii*). *D*, total vitreous protein from control and diabetic rats with Coomassie stain (*i*) and quantification (*ii*). *E*, total and mature IGF-2 in vitreous from control and diabetic rats (*i*) and quantification (*ii* and *iii*). *F*, vitreous IGFBP3 from control and diabetic rats (*i*) and quantification (*ii*). *G*, total vitreous IGF-1 from control and diabetic rats (*i*) and quantification (*ii*). All data analyzed by *t* tests. IGF-1, insulin-like growth factor-1; IGF-2, insulin-like growth factor-2.

Plasma insulin concentrations in control and age-matched diabetic animals are shown in Figure 4*A*. Serum IGF-2 content was examined by immunoblotting from sera of control and diabetic animals after 4 weeks of diabetes. Immunoblotting avoids interference by IGF binding proteins and reveals multiple molecular weight moieties (31). Figure 6*Ai* and *ii* is a representative immunoblot comparing serum from a P7 control rat and nondiabetic control and 4 weeks duration diabetic rats. Long exposure blots reveal low levels of mature IGF-2 in P7 rats (Fig. 6*Aii*), and the adult animals exhibit negligible mature IGF-2. By contrast, moderate levels of 22 kDa pro-IGF-2 are detected in adult sera which were unaltered by diabetes (Fig. 6*Aiii*). Retinas from a P7 pup exhibit <1 ng of mature IGF-2 but show 15 kDa “big” IGF-2, again with no change resulting from diabetes (Fig. 6*Bi* and *ii*). By contrast, 4 weeks of diabetes reduces serum content of mature IGF-1 (Fig. 6*Ci* and *ii*).

The intraocular concentrations of ligands were also measured in vitreous gel extracted from the same animals. Coomassie staining showed a 3-fold higher total protein content (by protein assay) (Fig. 6*Di* and *ii*) from the diabetic rat eyes despite loading equal volumes of vitreous, likely because of blood–retinal barrier leakage (32). Nevertheless, diabetes reduced total rat vitreous IGF-2 content by 78%, and mature IGF-2 by 80% (Fig. 6*Ei*, *ii* and *iii*). Total vitreous IGFBP3 content was reduced by 60%, especially the 30 kDa form (Fig. 6*Fi*, *ii* and *iii*). Total vitreous IGF-1 content was reduced by 50%, although diabetes increased a 17 kDa form (Fig. 6*Gi* and *ii*). In contrast to serum (Fig. 6*C*), rat vitreous does not contain detectable mature IGF-1 (7.6 kD) (Fig. 6*Gi*). In addition, rats with 4 weeks of diabetes and age-matched controls have equivalent serum IGF-2 content, whereas mature IGF-1 is dramatically reduced in the same animals (Fig. 6*C*). Thus, the reduction of serum and vitreous IGF-2 and IGF-1 by diabetes is specific relative to the overall increase in vitreous protein and not likely because of IGF binding proteins.

### Intraocular IGF-2 administration restores the retinal IR pathway in diabetic rats

**Figure 7:**
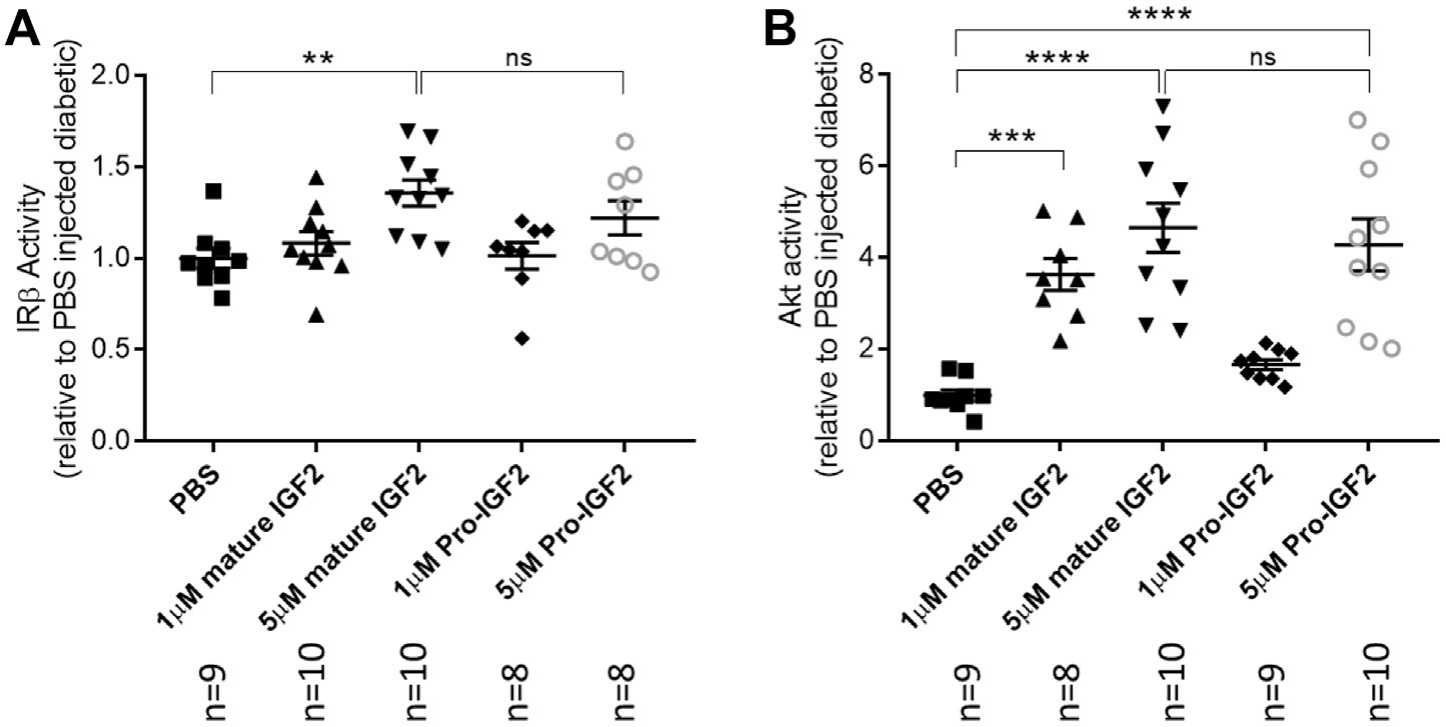
**Intravitreal stimulation of IRβ activities by mature IGF-2 in diabetic rat retina.** Mature or pro-IGF-2 or PBS was given as a single intravitreal injection (5 μl) in two concentrations to age-matched control and diabetic Sprague-Dawley rats after 12 weeks of diabetes. Retinas were harvested 60 min later and IR-ß kinase activity (*A*) and Akt1 kinase activity (*B*) were determined as described previously (11, 33). Data analyzed by one-way ANOVA (F (_4,40_) = 4.7, *p* = 0.0033 for IR-ß kinase activity (*A*) and F (_4,41_) = 15.83, *p* < 0.0001 for Akt1 kinase activity (*B*)) followed by *post hoc* Tukey's multiple comparisons test, with a single pooled variance [∗∗*p* = 0.0067 for IR-ß kinase activity (*A*) and ∗∗∗*p* = 0.0008 and ∗∗∗∗*p* < 0.0001 for Akt1 kinase activity (*B*)]. IGF-2, insulin-like growth factor-2; IR, insulin receptor.

Diabetes impairs the retinal IR signaling pathway and subconjunctival insulin injections restore signaling and normal rates of cell death and protein synthesis (11, 33). Therefore, we injected recombinant mature and pro-IGF-2 intravitreally in rats with diabetes. Both ligand forms caused rapid dose-dependent increases in retinal IR and Akt1 kinase activities (Fig. 7, *A* and *B*). These results demonstrate the ability to restore prosurvival signaling and cellular viability in the retina of insulin-deficient rodents.

## Discussion

The purpose of this study was to determine the basal regulation of adult retinal IR activity and how it is affected by insulin-deficient diabetes. The factors that regulate homeostasis of the postmitotic adult retina and how they are impaired by diabetes are incompletely known. Here we show for the first time (1) retinal IGF-2 transcript levels remain high in adult rats, suggesting an active role in homeostasis; (2) basal retinal IR kinase activity depends primarily on locally generated IGF-2 rather than insulin or IGF-1; (3) diabetes reduces retinal *IGF-2* mRNA content and IGF-2 protein content within vitreous fluid; and (4) intravitreal IGF-2 injection augments prosurvival IR kinase and Akt1 kinase activity in diabetic rat retinas. Together, these findings provide new understanding of the regulation of the retinal IR and how it may be altered by diabetes.

Previous work has shown that the retinal IR and downstream effects of Irs-2 are important for development of inner and outer retinal neurons and the optic nerve (3, 4, 5, 34, 35, 36), but the regulation of basal IR activity in adult mammals has not been fully determined. We previously showed that basal retinal IR kinase activity is significantly higher in the retina *versus* the liver and skeletal muscle of fasted adult rats and does not fluctuate with feeding and fasting (6). Moreover, portal vein injection of insulin in control rats increased IRß autophosphorylation of skeletal muscle but not retina (6). Insulin and IGF-2 both increase IR autophosphorylation in *ex vivo* retinas (6), but the control of basal kinase activity in intact eyes has not been tested previously. In this study, we employed multiple approaches to address the question. First, we found that retinal insulin concentration remains constant during feeding and fasting and during insulin-deficient diabetes induced by streptozotocin. These findings are consistent with those of LeRoith *et al.* (24) who reviewed multiple studies showing similar insulin concentrations in brain and other tissues, and a lack of variation with fasting or streptozotocin administration. Therefore, CNS IR activity is regulated differently from that of peripheral tissues such as liver, skeletal muscle, and adipose and raises the possibility that other ligands contribute to the high basal retinal IR activity.

Next, we found that 1% serum from insulin-deficient rats failed to stimulate IR autophosphorylation in R28 retinal neuronal cells, in contrast to serum from fed or fasted rats. Serum from animals subjected to diabetes and fasting exhibit distinctive profiles, despite the fact both conditions are characterized by low serum insulin concentrations and both conditions ultimately induce skeletal muscle and fat breakdown. Further, immunodepletion of IGF-2 but not albumin or IGF-1 from the serum reduced the ability to stimulate IR and IGF1R autophosphorylation by half, and there was no additive effect of dual IGF-2 and IGF-1 depletion. IGF-2 and insulin both induced concentration-dependent increases of IR-ß and IGF-1ß-PY immunoreactivity. Of note, these effects are seen in cells from which the 10% FBS was removed to reduce basal IR PY. Thus, the stimulated effects are distinct from the *in vivo* environment in which the IR kinase activity and autophosphorylation are already high. Nevertheless, they support a specific role of IGF-2 to activate the IR from retinal cells.

A role for IGF-2 in the adult retina is further supported by persistent expression of *IGF-2* mRNA in the normal adult rat retina, whereas liver *IGF-2* gene expression markedly declines after birth. Adult rat retinas continue to express high levels of *IGF-2* and minimal *INS* mRNA. These findings are in agreement with the finding by Lofqvist *et al.* (16) that the retina expresses 100 to 1000 fold more *IGF-2* than *INS* mRNA. They are also consistent with persistent high expression of *IGF-2* in multiple adult brain regions, notably hippocampus and neural stem cells (37, 38) because of increased expression from the maternal allele (22). In fact, retina has one of the highest levels of *IGF-2* mRNA expression in humans, second only to the placenta (https://www.proteinatlas.org/ENSG00000167244-IGF2/tissue).

Further, the *in vivo* role of IGF-2 was confirmed *via* the use of intravitreally injected antibodies to insulin, IGF-1, and IGF-2 in normal rats. The IGF-2 antibody recognizes only IGF-2 by Western blotting. Remarkably, only IGF-2 antibodies decreased basal IR kinase activity in repeated experiments, and the 20% reduction in activity is equivalent to the change seen in diabetic rats (9). Residual IR activity could be due to light exposure in photoreceptors (39) or limited ability of the antibody to penetrate the retina. These findings do not eliminate the possibility that small amounts of insulin or IGF-1 may contribute to retinal IR basal activity. The exclusive presence of the exon 11-form of *Insr* mRNA in the retina, as well as the brain, is consistent with a dominant role for IGF-2 and suggests a fundamentally different means of IR regulation in the nervous system *versus* peripheral tissues that fluctuate with nutrient status. LeRoith *et al.* (2, 24) have taught that the insulin/IGF system originated evolutionarily in the CNS of mollusks, insects, and in bacteria before the development of the pancreas. Presumably, most brain functions require stable growth factor control of homeostasis compared with peripheral tissues (skeletal muscle, liver, adipose) that respond to fluctuating nutrient intake, so locally expressed IGF-1 and/or IGF-2 acting in an autocrine or paracrine manner would provide needs of the retina for anabolic and cell survival signals rather than circulating insulin. Collectively, the data indicate that the retinal IR is activated by both ligand-dependent and ligand-independent, light-induced mechanisms. The latter is primarily related to photoreceptors (40), whereas the ligand-dependent activation is more likely acting on nonphotosensitive inner retinal cells.

After establishing IGF-2 as the likely key IR ligand, we next examined the expression patterns of IGF-1 and IGF-2 protein in postnatal animals. We employed an immunoblotting method described by Qiu *et al.* (31) which facilitates resolution of multiple molecular weight forms of IGF-2 that cannot be accomplished by radioimmunoassay or enzyme-linked immunosorbent assay methods. Analysis of serum shows prominent 22 kDa pro-IGF-2 in adult rats without detectable smaller forms. Rat retina exhibited prominent 25 and 22 kDa pro-IGF-2 bands and a less prominent 15 kDa “big” band, but multiple experiments failed to detect any 7.5 kDa mature IGF-2 protein in the retina. Diabetes did not alter the content or pattern of IGF-2 in the retina. By contrast, immunoblotting showed marked reduction of the mature IGF-1 peptide in response to diabetes.

The absence of detectable mature IGF-2 protein in the adult rat retina could result from rapid receptor binding and internalization (41) and prompted us to examine the intraocular vitreous fluid. Although the total vitreous protein concentration was 3-fold greater in diabetic animals than controls, the total and mature IGF-2 immunoreactivity was reduced by nearly 80%. Diabetes also reduced the vitreous IGF-binding protein 3 content by approximately 60%, so changes in binding proteins likely do not account for the reduction of IGF-2. IGF-1 content was reduced in the vitreous similar to IGF-2.

Functional activation of the retinal IR was then examined. Basal autophosphorylation of IR-ß and IGF1Rß revealed distinctly greater immunoreactivity of IR-ß than IGF1Rß in retina and prominent IR-ß but no detectable IGF1Rß activation in liver. We also found that intravitreal injection of recombinant Des[1–6] IGF-2 preferentially increased retinal IR-ß activity compared with IGF1Rß. In *ex vivo* retinas both recombinant mature and pro-IGF-2 increased Akt activity in a PI-3-kinase dependent manner. Thus, basal retinal IR activity may be influenced by both pro and mature forms of IGF-2.

Pancreas-derived insulin regulates systemic homeostasis, notably substrate uptake and protein and lipid synthesis, in skeletal muscle and adipose tissue, and insulin deficiency causes rapid atrophy of skeletal muscle and fat (42). In contrast, chronic caloric starvation, with reduced serum insulin concentrations (43) is not known to cause a diabetes-like retinopathy. Further evidence that pancreas-derived insulin is not the predominant retinal IR ligand includes the finding that retinal insulin content does not vary with feeding and fasting or from insulin-deficient diabetes, in spite of very low serum levels and high retinal IR-ß activity in fasted animals (6). Thus, we tested the roles of IGF-1 and IGF-2 and found that intravitreal administration of IGF-2 antibodies, but not insulin or IGF-1 antibodies, decreased basal retinal IR kinase activity in normal rats. The finding that retina expresses only the exon-11 minus splice variant of the IR which has high affinity for IGF-2 (6, 30, 44) further supports a functional role of IGF-2 in the normal retina. We previously used subconjunctivally administered insulin to restore IRß kinase activity in diabetic rat retinas (11, 33, 45), but the data described in this report suggest that IGF-2 rather than insulin may be more physiologically relevant to sustain prosurvival kinase activity and potentially ameliorate diabetic retinal sensory neuropathy. IGF-2 mRNA content is significantly reduced in the sciatic nerve of diabetic rats (46), and sciatic nerve expresses exclusively the exon 11-IR splice variant (47). Thus, loss of IGF-2/IR-dependent neurotrophic support could be a common feature of sensory neuropathies in diabetes. Collectively, this information is important to understand the metabolic and cell survival consequences of the high basal IR activity in normal retina and efforts to retinal function and vision in diabetes.

## Experimental procedures

### Animals

All experiments were conducted in accordance with the Association for Research in Vision and Ophthalmology Resolution on the Care and Use of Laboratory Animals and approved by the University of Michigan IUCAC. Rats with starting weights 170 to 200 g (Fig. S1*A*) were housed under 12-h light/dark cycles and had free access to a standard rat chow and water, and retinas were routinely harvested under room light conditions between 9 and 11 AM. Thus, photoreceptor insulin receptors were probably already stimulated by light as shown by Rajala *et al.* (48). Diabetes was induced by intraperitoneal injection of streptozotocin (65 mg/kg; Sigma) dissolved in 10 mM sodium citrate buffer, pH 4.5, and control rats received equivalent volumes of buffer alone as described previously (33). Streptozotocin-injected rats were considered diabetic when exhibiting blood glucose levels >13.9 mmol/l (250 mg/dl) within 5 days after diabetes induction (Accu-Check Nano; Roche) (Fig. S1*B*). Body weights and blood glucose levels were monitored weekly. Some animals were evaluated after ad lib feeding compared with a 16 h overnight fast. At the time of isolation of the retinas, the rats were anesthetized with inhaled isoflurane and euthanized by decapitation following motor reflex loss for rapid dissection of retinas, brains, and livers. Tissues were immediately frozen in liquid nitrogen and stored at −80 °C until analysis.

### Rat vitreous preparation

Rat vitreous was removed from enucleated eyes, placed on ice, spun 17*g* for 5 min at +4 °C, and the supernatant frozen at −80 °C.

### Immunoprecipitation and immunoblotting

IGF-2 was quantified by immunoblotting *via* the method described by Qiu *et al.* (31). This method provides the ability to assess the individual isoforms of IGF-2, whereas radioimmunoassay or enzyme-linked immunoassays do not. Briefly, retinas were lysed IP Buffer (50 mM Hepes, 137.5 mM sodium chloride, 2 mM sodium orthovanadate, 10 mM sodium pyrophosphate, 10 mM sodium fluoride, 2 mM EDTA, 2 mM PMSF, 0.1566% benzamidine, 10% glycerol, 1% NP-40, and one mini EDTA-free protease inhibitor tablet (Roche) per 10 ml buffer), and proteins were separated by SDS-PAGE using the Bolt system (Thermo Fisher Scientific) and transferred to nitrocellulose using the iBlot system (Thermo Fisher Scientific), then treated with Miser antibody extender solution (Thermo Fisher Scientific). Mouse anti-rat IGF-2 monoclonal antibody (clone S1F2; Millipore Sigma) was used as a primary antibody. Immunoblots were developed with the Amersham ECL Select reagent (GE Healthcare Bio-Sciences) according to the manufacturer’s instructions.

All antibodies were purchased and used according to the manufacturer’s suggestions. Anti-IRβ (sc-711) and IGF-IRβ (sc-713) were from Santa Cruz Biotechnology; anti-PY was from Millipore Sigma (05-321, clone 4G10); anti-phosphoSer473 and anti-Akt1 and anti-Akt3 and anti-total Akt, anti-phospho and total p44/p42 MAPK, anti-phosphoSer9 and total GSK3beta, anti-phosphoThr389 and total P70S6K, and anti-phosphoSer2448 and total mTOR were from Cell Signaling Technology. For immunoprecipitations, GammaBind Protein G Sepharose beads were purchased from GE Healthcare Bio-Sciences. For IGF-2 detection, the anti-mouse IgG horseradish peroxidase (HRP)–conjugated secondary antibodies from Promega were used. For detection, other proteins secondary antibodies from GE Healthcare Bio-Sciences were used: anti-rabbit IgG HRP-linked secondary antibodies (NA934) or anti-mouse IgG HRP-linked secondary antibodies (NA931). Recombinant human Des[1–3]-insulin-like Growth Factor-I (Des[1–3]-IGF-1) (DU100, GroPep) and recombinant human Des[1–6]-insulin-like growth factor-II (Des[1–6]-IGF-2) (MU100, GroPep) were used as a loading control for mature forms.

### Immunodepletion studies

R28 cells were seeded with 2 × 10^6^ cell density for overnight incubation in 60 mm dishes. The next day, cells were washed once with Dulbecco's phosphate-buffered saline and starved in Dulbecco's modified Eagle's medium (DMEM) media-free serum for 3 h before processed with prewarmed DMEM media immunodepleted under different conditions for 5 min at 37 C in CO_2_ incubator. The immunodepletion studies were performed in DMEM media with 1% rat serum and 0.1% BSA after pretreatment with Protein G sepharose (GE Healthcare, 17-0618-01) for 30 min at 37 C. Protein G sepharose was pretreated before use in PBS with 0.1% BSA for 30 min at 37 C. For immunodepletion, 1.2 ml of pretreated DMEM media with 10 μl of different antibodies to anti-rat serum albumin (Abcore Inc, rabbit, AC20-0302), anti-human insulin (Sigma, I2018, clone K36aC10), anti-IGFBP3 (Santa Cruz, sc-9028, clone H-98), anti-IGF-1 (EMD Millipore, 05-172, clone Sm1.2), anti-IGF-2 (EMD Millipore, 05-166, clone S1F2) for 1 h at 37 C were used. Anti-human insulin antibody reacts with rat, human, and bovine insulin. Anti-IGF-1 and anti-IGF-2 antibodies recognize both rat and human antigens. There was no cross reactivity between IGF-1 and IGF-2 on Western blotting.

As a control for the immunodepletion studies, we used serum-free DMEM media and DMEM containing 1% rat serum with 0.1% BSA. To assess the efficiency of immunoprecipitations, we immunoprecipitated bovine insulin in different concentrations in the DMEM media in the same conditions to other antigens with different antibodies. Serum free DMEM media with 0.1% BSA and bovine insulin or recombinant human mature Des[1–6]-IGF-2 without immunodepletion were controls for PY IR-ß and IGF-1ßR in R28 cells.

We use pretreated with Protein G sepharose to pull down antibody complexes with antigens. The supernatant of the immunodepleted 1% rat serum DMEM media were used in experiment to assess its ability to phosphorylate IR and IGF1R in R28 cells after immunodepletion.

### RNA preparation, cDNA amplification, and quantitative RT-PCR

Total RNA isolated using QIAshredder and RNeasy Plus Mini Kit (both from Qiagen), cDNA was made with blend of oligo(dT) and random hexamer primers and iScript cDNA synthesis kit (Bio-Rad Laboratories). Quantitative real-time PCR was performed with cDNA using a TaqMan Universal PCR Master Mix (Thermo Fisher Scientific) on the real-time C1000 thermal cycler CFX384 (Bio-Rad). Primer/probes used for analysis were *Igf1* (Rn00710306_m1), *Igf2* (Rn01454518_m1), *Igf1r* (Rn00583837_m1), *Insr* (Rn00567070_m1), and *Actb* (Rn00667869_m1), all from Thermo Fisher Scientific. Each sample was normalized to β-actin.

### Intraocular injections

Monoclonal antibodies against insulin, IGF-1, and IGF-2 were injected intravitreally of control rats in a volume of 8.2 μl. The antibody specificities were verified with a combination of recombinant proteins and tissues with high levels of ligand expression, brain, and pancreas. Retinas were harvested 1 h after injection and flash frozen. Animals were harvested 30 min postinjection, and retinas were assayed for IR and Akt1 kinase activities.

Five microliter of 1 μM Des[1–6]-IGF-2 (MU100 – mature, GroPep) or 5 μl PBS were injected intravitreally in control male Sprague-Dawley rat, and retinas were harvested 30 min later.

*Ex vivo* rat retinas were treated with 10 nM or 100 nM of receptor grade recombinant human insulin-like growth factor-II (Des[1–6]-IGF-2) mature or pro-form (pro-rhIGF-2), (MU100 – mature and AHU020 – pro-rhIGF-2, GroPep) for 5 min with or without LY294002 (50 μM, 15 min), then lysates were analyzed for phosphor-Ser473Akt and total Akt.

### Insulin assays

Insulin concentrations from serum, vitreous, and retina were quantified with the Ultrasensitive Rat RIA Kit (SRI-13K; Millipore Sigma) according to manufacturer’s instructions and as previously described (11).

### Kinase assays

Retinal insulin receptor kinase and Akt1 kinase activities were quantified in retinal lysates as described previously (11, 33). Retinas were sonicated in lysis buffer, and 500 μg of tissue lysates were immunoprecipitated using anti-IRß (Santa Cruz) or anti-Akt1 (Millipore) antibodies. After washing the immune complexes, specific kinase activity was assessed using radiolabeled ATP (25 mCi/ml [^32^P]-ATP, Amersham) incorporation onto the respective peptides (poly Glu:Tyr for IRß and crosstide (GRPRTSS-FAEG for Akt1)). Equivalent efficiency of the immunoprecipitation was verified by immunoblot analysis of the immune complexes after decay of the radioactivity.

### Statistical analysis

Statistical analyses between two groups were done by Student’s *t* tests, with two-tailed *p* values, where *p* < 0.05 (n ≥ 3) set as significant. For multiple-group comparison, analyses were performed using one-way ANOVA when multiple variables were compared followed by *post hoc* Tukey's multiple comparisons test with a single pooled variance. Statistical analyses were performed using GraphPad Prism 8 software (GraphPad Software).

## Data availability

All data described are contained in the manuscript.

## Supporting information

This article contains supporting information.

## Conflict of interest

The authors declare that they have no conflicts of interest with the contents of this article.
