# Supplementary material for: Insulin-like growth factor-2 regulates basal retinal insulin receptor activity
Source: J Biol Chem. 2021 Apr 26;296:100712. doi: 10.1016/j.jbc.2021.100712 (PMC8138762; doi:10.1016/j.jbc.2021.100712)
Supplement: Supplemental Figure S1 [file mmc1.pdf]

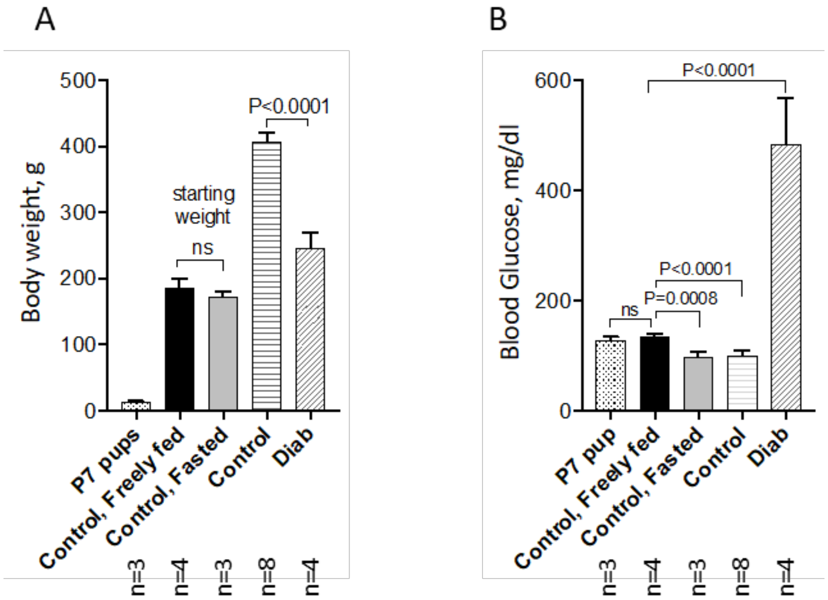

Figure S1 Rat characteristics.

Body weight (A) and blood glucose levels (B) in 7-day old pups, fasted or fed adults, and streptozotocin-induced diabetic male Sprague-Dawley rats and their age-matched non-diabetic controls. Data analyzed by an ordinary one-way ANOVA ($F_{(4,17)} = 470.9$, $p<0.0001$ for body weight (A) and $F_{(4,17)} = 86.93$, $p<0.0001$ for blood glucose levels (B)) followed by *post hoc* Tukey's multiple comparisons test. Error bars, SD.
